# Commentary: Trunk Muscle Activity during Drop Jump Performance in Adolescent Athletes with Back Pain

**DOI:** 10.3389/fphys.2018.00298

**Published:** 2018-04-16

**Authors:** Thorvaldur S. Palsson, J. P. Caneiro, Rogerio Pessoto Hirata, Derek Griffin, William Gibson, Mervyn J. Travers

**Affiliations:** ^1^Department of Health Science and Technology, Sanse-Motorisk Interaktion (SMI), Aalborg University, Aalborg, Denmark; ^2^School of Physiotherapy and Exercise Science, Curtin University, Perth, WA, Australia; ^3^Body Logic Physiotherapy Clinic, Perth, WA, Australia; ^4^Bon Secours Hospital, Tralee, Ireland; ^5^School of Physiotherapy, The University of Notre Dame Australia, Fremantle, WA, Australia

**Keywords:** adolescent back pain, pain-related fear, trunk stability, negative beliefs in back pain, management of back pain

It was with great interest we read the recently published article “*Trunk Muscle Activity during Drop Jump Performance in Adolescent Athletes with Back Pain.”* Investigating back pain (BP) in adolescents is commendable as there is growing evidence that for many, an experience of BP as early as 14 years of age may relate to ongoing pain in adulthood (Coenen et al., 2017). Indeed, the conventional narrative is changing as individual physical factors such as posture, use of schoolbags, and hypermobility are only weakly associated with adolescent BP. Rather, factors which predict BP at a young age are considered to be multi-dimensional and include gender, negative BP beliefs and poor mental health (O'Sullivan et al., 2017; Smith et al., 2017). Mueller et al. (2017) have focused on a single physical factor (trunk muscle activation patterns) drawing inferences regarding BP prevention and treatment. This article prompts consideration of three essential aspects regarding research design and interpretation of findings:

1. Interpreting results from cross-sectional designs
2. Interpreting pain-related differences in motor behavior
3. Translating and conveying scientific results to the end-user (patients, healthcare professionals and policy makers).

## How should results from cross-sectional designs be interpreted?

The results of Mueller et al. (2017) suggest an altered neuromuscular activation pattern (increased trunk muscle activity during drop landing) in adolescent athletes with BP. Their study recruited 11 adolescent athletes with BP and 11 controls into a cross-sectional study. Subjects conducted 3 drop jumps onto a force plate whilst trunk muscle activity was recorded via electromyography (EMG). The EMG data was analyzed via multiple comparisons (un-paired *t*-tests) over six muscles without any attempt to correct for multiple comparisons, questioning the reported statistical difference. Such limitations should be acknowledged and taken in consideration when interpreting the results.

Statistical issues aside, it is problematic when a cross-sectional study design (which can offer nothing more than association) is used to imply both causality and treatment recommendations for BP. In the presence of only cross-sectional data and an absence of key known confounding variables, the conclusion “*For prevention and therapy, specific sensorimotor exercises addressing the transverse trunk muscles with e.g., 3-dimensional loading situations might be beneficial”* (Mueller et al., 2017) is speculative. While cross-sectional data are useful in identifying associations, inferences regarding causality should not be made from these study designs (Stovitz et al., 2017). The mere presence of two findings at the same time does not mean they are related. For example, a recent and highly publicized retrospective cohort study has demonstrated concussion risk to be *co-incidentally* associated with non-plausible predictor variables (altitude and team logo) (Smoliga and Zavorsky, 2017).

## How should pain-related differences in motor behavior be interpreted?

This study demonstrated differential motor behavior (increased trunk muscle activity during drop landing) in adolescents with BP. Thus, it contributes to a substantial existing body of research that has consistently shown that pain is associated with increased trunk muscle activity (Arendt-Nielsen et al., 1996; Graven-Nielsen et al., 1997; Hodges et al., 2009; Wong et al., 2016). However, Mueller et al. (2017) posit that the observed increased trunk muscle activity may be a maladaptive strategy that needs correction without considering that such phenomena may occur as a consequence of an interplay of multidimensional factors, recently reported to be related to BP experience in adolescents (Smith et al., 2017).

The current understanding of pain suggests that it is an emergent protective output in response to an implicit evaluation of danger (Moseley, 2007; Tabor et al., 2016; Moseley and Butler, 2017), aimed at minimizing the real or perceived risk of further pain or damage (van Dieen et al., 2017). Thus, it is entirely possible that the observed motor behavior may reflect a helpful, top-down adaptive strategy when faced with a high-load task such as jumping; potentially perceived as threatening when one has a sore back. Critically, perceiving a task as threatening has been related to fear of movement, pain intensity (Karayannis et al., 2013; Wallwork et al., 2016) and changes in distribution of activity within (Tucker et al., 2009) and between muscles (Moseley et al., 2004). Further, cognitive factors such as pain catastrophizing can influence trunk muscle activation in both experimentally-induced (Ross et al., 2017) and clinical BP (Pakzad et al., 2016). This well-established interplay of cognition, emotions and behavior (Vlaeyen et al., 2016) was not acknowledged in the current paper. Thus, assigning meaning to differential motor behavior may be incomplete and speculative. Therefore, it is inappropriate and unfounded to recommend interventions that aim to correct the observed motor behavior (core stability training).

## How should this be translated and conveyed to the end-user?

By suggesting the current findings are causally related to development and treatment of BP in adolescents, the authors then advocate for an approach to management consisting of “*sensorimotor exercises”* without specifying what such a program would/should consist of. Neither do they suggest a biologically plausible rationale for such an intervention, considering that such approaches (e.g., core stability exercises) seem no more effective than general exercise (Smith et al., 2014) in both acute (Macedo et al., 2016) and chronic (Saragiotto et al., 2016) BP. In fact, evidence suggests that improvements in BP are unrelated to motor changes in trunk muscles (Mannion et al., 2012). Rather, improvements are related to development of helpful back beliefs, reducing pain-related catastrophic thoughts, about the need to protect the body (Mannion et al., 2012), and improved self-efficacy (Bunzli et al., 2016).

When examined through the lens of treatment effect, core stability training may be considered benign. However, it is important to consider the potential harm from perpetuation of biomedically-based explanations for BP (Darlow et al., 2013; Bunzli et al., 2017). Motor responses should be considered behaviors; and like any behavior, they are individual and influenced by a range of factors, including (but not limited to) physical, cognitive and emotional factors. Therefore, higher levels of muscle activity may represent one of many contributors to ongoing pain, highlighting that clinicians may need to have a broad scope in terms of assessment and treatment strategies. Recent, qualitative studies demonstrate (Darlow et al., 2013; Bunzli et al., 2017) the importance of and need for caution in communicating about BP; in particular how negative views about the back can be pervasive in society. Researchers must carefully consider how reporting of results might be interpreted by clinicians and people with BP, as research recommendations can *misguide* clinical practice. To ensure accuracy in research outcomes, study designs and analyses should reflect the research questions and be reported accordingly. Furthermore, interpretation of results should not extend beyond the limits of the research design to ensure useful dissemination of findings.

## Author contributions

TP and MT as first and last authors lead the work on the paper and made the first draft. JC and WG made significant contributions to the focus of the manuscript. RH provided feedback and contribution to the statistical section and metodological part in the study. DG contributed to the clinical aspects and utility of the findings from a clinical perspective.

### Conflict of interest statement

The authors declare that the research was conducted in the absence of any commercial or financial relationships that could be construed as a potential conflict of interest.
